# Two-Dose Varicella Vaccination Coverage Among Children Aged 7 years — Six Sentinel Sites, United States, 2006–2012

**Published:** 2014-02-28

**Authors:** Adriana S. Lopez, Cristina Cardemil, Laura J. Pabst, Karen A. Cullen, Jessica Leung, Stephanie R. Bialek

**Affiliations:** 1 Division of Viral Diseases; 2 Immunization Services Division, National Center for Immunization and Respiratory Diseases, CDC; 3 Division of Parasitic Diseases and Malaria, Center for Global Health, CDC

In 2007, the Advisory Committee on Immunization Practices (ACIP) recommended a routine second dose of varicella vaccine for children at age 4–6 years, in addition to the first dose given at age 12–15 months (1). One strategy recommended for increasing varicella vaccination coverage is a school entry requirement of proof of varicella immunity (1,2). To determine the extent of implementation of the routine 2-dose varicella vaccination program, the number of states with a 2-dose varicella vaccination elementary school entry requirement in 2012 was compared with the number in 2007, and 2-dose varicella vaccination coverage during 2006 was compared with coverage in 2012 among children aged 7 years, using data from six Immunization Information System (IIS) sentinel sites. The number of states (including the District of Columbia) with a 2-dose varicella vaccination elementary school entry requirement increased from four in 2007 to 36 in 2012. Two-dose varicella vaccination coverage levels among children aged 7 years in the six IIS sentinel sites increased from a range of 3.6%–8.9% in 2006 to a range of 79.9%–92.0% in 2012 and were approaching the levels of 2-dose measles, mumps, and rubella (MMR) coverage, which had a range of 81.9%–94.0% in 2012. These increases suggest substantial progress in implementing the routine 2-dose varicella vaccination program in the first 6 years since its recommendation by ACIP. Wider adoption of 2-dose varicella vaccination school entry requirements might help progress toward the *Healthy People 2020* target of 95% of kindergarten students having received 2 doses of varicella vaccine.

Data on the number of states with 1-dose and 2-dose varicella vaccine elementary school entry requirements at the start of the school year were obtained from state immunization websites for 2007 and 2012. Data on varicella vaccination coverage were obtained from six sentinel IIS sites. IIS, also known as immunization registries, are computerized, population-based systems that consolidate data from participating vaccine providers and provide tools for supporting effective immunization strategies at the vaccination provider and program levels (3). The IIS sentinel site project is a collaboration between CDC and state- and city-based IIS. To be eligible to compete for CDC sentinel site funding, ≥85% of vaccination providers must participate in the IIS, ≥85% of children aged <19 years must have at least two vaccinations recorded in the IIS, and ≥70% of doses administered must be reported to the IIS within 30 days of administration. The six IIS sentinel sites funded for the 2013–2017 project period are Michigan, Minnesota, North Dakota, New York City, and Wisconsin, which include data from the entire jurisdiction, and Oregon, which includes data from six counties (56% of the state population).

De-identified individual record-level data were received from IIS sentinel sites and processed in accordance with IIS best practices (4). Children who were designated in the IIS as permanently inactive (i.e., deceased) or “moved or gone elsewhere” were excluded from analysis.

Varicella and MMR vaccination coverage were assessed at age 7 years to allow time for the 2-dose series to be completed. Two-dose varicella vaccination coverage estimates were calculated for each year of the study period (i.e., January 1, 2006–December 31, 2012) among children aged 7 years (born during January 1, 1999–December 31, 2005). Intercensal population estimates for 2006–2009 and postcensal estimates for 2010–2012 were used for the denominators (5). Valid doses of varicella vaccine were defined as dose 1 administered no earlier than 4 days before age 12 months, dose 2 administered at least 28 days after dose 1, and either dose administered on the same day as or ≥4 weeks after any other live vaccine.* Coverage was calculated by dividing the number of children aged 7 years with 2 valid doses of varicella vaccine by the U.S. Census estimate of the total number of same-aged children in the sentinel site population. To have a single measure of coverage at the six sites that could be compared from year to year, the unweighted average of the estimates for each of the six sites was calculated for each year.

Two-dose varicella vaccination coverage estimates derived from IIS data for children aged 6 years were compared with data from the kindergarten vaccination assessment for the four sentinel sites (Michigan, Minnesota, North Dakota, and Wisconsin) that had 2-dose varicella vaccination school entry requirements for the 2012–13 school year (6). Kindergarten assessments, conducted annually by federal immunization grantees through a vaccination coverage survey or census of enrolled students to determine compliance with school vaccination requirements (6), are the only available source of national data on 2-dose varicella vaccination coverage. Differences between 2-dose varicella vaccination coverage in 2012 at sites with and without 2-dose school entry requirements for children aged 6 years were examined and analyzed for statistical significance using the Wilcoxon-Mann-Whitney test.

The number of states requiring 2 doses of varicella vaccine for school entry increased rapidly, from four in 2007 to 36 by 2012, and all but one state required 1 or more doses of varicella vaccine for elementary school entry by the 2012–13 school year (Figure 1).

Varicella vaccination coverage levels with 2 doses among children aged 7 years increased greatly at the six IIS sentinel sites, from a range of 3.6%–8.9% in 2006 to a range of 79.9%–92.0% in 2012, approaching that of 2-dose MMR vaccination coverage, which ranged from 81.9% to 94.0% in 2012 (Figure 2). Implementation of the 2-dose varicella vaccination recommendation was rapid, with the average of coverage percentages increasing to 72.4% by 2009.

Coverage estimates for 2 doses of varicella vaccine among children aged 6 years at four IIS sites based on IIS data were similar to those reported in the kindergarten assessment. The IIS estimate was lower than the kindergarten assessment at two of the sites (percentage-point differences of 0.5 and 15.6) and higher at two sites (percentage-point differences of 2.0 and 4.4) (Table). Two-dose varicella vaccination coverage in 2012 for children aged 6 years was slightly higher in the four states with 2-dose school entry requirements (Michigan, Minnesota, North Dakota, and Wisconsin), compared with sites with only a 1-dose school entry requirement (New York City and Oregon), although this difference was not statistically significant (p=0.5) (Table).

## Editorial Note

During the first 6 years of the 2-dose varicella vaccination program, the number of states with 2-dose varicella vaccination elementary school entry requirements increased from four to 36, and 2-dose coverage among children aged 7 years in IIS sentinel sites increased from 4%–9% to 80%–92%, approaching the level for 2-dose MMR coverage. The rapid increase in 2-dose coverage after the ACIP recommendation and before 2-dose school entry requirements were widely adopted suggests extensive implementation of the recommendation by health-care providers. School entry requirements have been useful for increasing 1-dose varicella vaccination coverage among children (2). Adoption of 2-dose varicella vaccination school entry requirements by additional states and for higher grades might help reach the *Healthy People 2020* targets of 95% and 90% 2-dose coverage among kindergarten and adolescent students, respectively.

IIS sentinel sites provide an important source of population-based, provider-verified vaccination data and can be useful for assessing coverage for vaccines, such as varicella, for which other mechanisms to estimate coverage nationally are inadequate. Two-dose varicella vaccination coverage data are available from surveys of kindergarten-aged children; however, data collection and validation methodologies vary by state, and data are limited to doses required for school entry. Two-dose varicella vaccination coverage estimates for the 2012–13 school year based on IIS data were similar to those obtained from the kindergarten assessment, except for one site (6). Improvements in kindergarten survey methodology and ongoing adoption of 2-dose varicella school entry requirements will make it increasingly feasible to estimate 2-dose coverage for varicella vaccination nationally using data from kindergarten students, as is already done for MMR coverage.

The findings in this report are subject to at least two limitations. First, census-based denominators were used, which might have resulted in underestimation of varicella protection because children with a history of varicella disease are included in the denominator even though varicella vaccination would not be indicated for them. Second, the IIS sentinel sites are highly selected and might not be representative of other cities and states.

The 1-dose varicella vaccination program, implemented in 1996, resulted in 70%–90% declines in varicella disease incidence, hospitalizations, and mortality (7–9). The routine 2-dose varicella vaccination program was implemented to further decrease varicella disease and control outbreaks. Since its implementation in 2007, declines in varicella incidence and outbreaks ranging from 67% to 76% have been reported (10). Further declines in varicella incidence and outbreaks might occur as higher 2-dose varicella vaccination coverage is achieved.

What is already known on this topic?

A second dose of varicella vaccine was recommended for children by the Advisory Committee on Immunization Practices in 2007, and the recommendation has been followed by decreases in varicella incidence nationwide. However, estimates of 2-dose varicella vaccination coverage have not been available previously.

What is added by this report?

The number of states with a 2-dose varicella vaccine elementary school entry requirement increased from four in 2007 to 36 in 2012. Two-dose varicella vaccination coverage levels among children aged 7 years in six selected sentinel sites increased from a range of 3.6%–8.9% in 2006 to a range of 79.9%–92.0% in 2012, approaching the coverage level for 2 doses of measles, mumps, and rubella vaccine.

What are the implications for public health practice?

Health-care providers have been important to the increase in coverage levels for 2 doses of varicella vaccine. Wider adoption of 2-dose varicella vaccine school entry requirements in more states and higher grades might help reach the *Healthy People 2020* targets of 95% and 90% 2-dose varicella vaccination coverage among kindergarten and adolescent students, respectively.

## Figures and Tables

**FIGURE 1:**
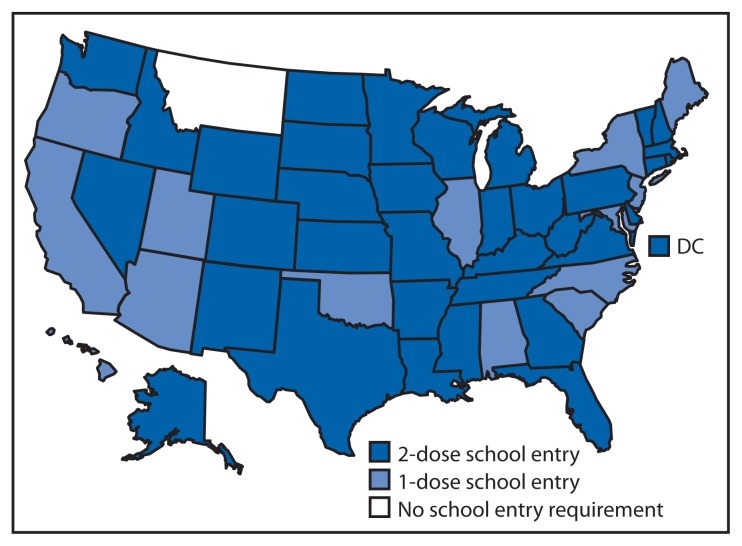
Varicella vaccine school entry requirements, by number of doses required — United States, September 2012

**FIGURE 2:**
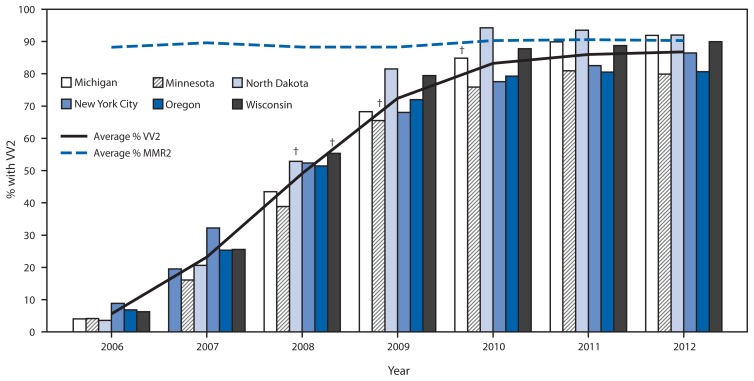
Among children aged 7 years, average percentage of 2-dose varicella vaccination coverage (VV2), compared with average percentage of 2-dose measles, mumps, and rubella (MMR2) vaccination coverage, and VV2 by Immunization Information System (IIS) sentinel site^*^ — six IIS sites, United States, 2006–2012 ^*^ Data for 3,633,391 children aged 7 years for the period 2006–2012 were analyzed to estimate VV2. The average number of children available for analysis per sentinel site during that period ranged from 10,343 in North Dakota to 159,167 in New York City. ^†^VV2 became required for elementary school entry in 2008 in North Dakota and Wisconsin, in 2009 in Minnesota, and in 2010 in Michigan.

**TABLE:** Two-dose varicella vaccination coverage among children aged 6 years, based on 2012 Immunization Information System (IIS) data, compared with 2-dose varicella vaccination coverage based on a 2012–13 kindergarten school year survey — IIS sentinel sites, United States

Sentinel site	2-dose varicella vaccination coverage, 2012 IIS data %	2-dose varicella vaccination coverage, 2012–13 kindergarten school year survey %
**Sites requiring 2 doses of varicella vaccine for school entry** *		
Michigan	92.2	92.9
Minnesota	80.3	95.9
North Dakota	92.9	88.5
Wisconsin	93.1	91.1
**Sites requiring 1 dose of varicella vaccine for school entry** *		
New York City	89.1	—
Oregon	80.9	—
**Average % for all six sites**	**88.1**	**—**

* The differences in 2-dose varicella vaccination coverage among sites requiring 2 doses and sites requiring 1 dose were not statistically significant (p=0.5).

## Footnotes

* Additional information available at http://www.cdc.gov/vaccines/programs/iis/interop-proj/cds.html.

## References

[1] CDC (2007). Prevention of varicella: recommendations of the Advisory Committee on Immunization Practices (ACIP). MMWR.

[2] CDC. Advisory Committee on Immunization Practices (ACIP) (2006). General recommendations on immunization: recommendations of the Advisory Committee on Immunization Practices (ACIP). MMWR.

[3] Community Preventive Services Task Force (2010). Universally recommended vaccinations: immunization information systems. Guide to community preventive services.

[4] Williams W, Lowery NE, Lyalin D (2011). Development and utilization of best practice operational guidelines for immunization information systems. J Public Health Manag Pract.

[5] US Census Bureau (2012). State single year of age and sex population estimates: April 1, 2010 to July 1, 2012—resident.

[6] CDC (2013). Vaccination coverage among children in kindergarten—United States, 2012–13 school year. MMWR.

[7] Guris D, Jumaan AO, Mascola L (2008). Changing varicella epidemiology in active surveillance sites—United States, 1995–2005. J Infect Dis.

[8] Marin M, Zhang JX, Seward JF (2011). Near elimination of varicella deaths in the US after implementation of the varicella vaccination program. Pediatrics.

[9] Lopez AS, Zhang J, Brown C, Bialek S (2011). Varicella-related hospitalizations in the United States, 2000–2006: the 1-dose varicella vaccination era. Pediatrics.

[10] Bialek SR, Perella D, Zhang J (2013). Impact of a routine 2-dose varicella vaccination program on varicella epidemiology. Pediatrics.

